# Practical Observations in Typhoid Fever

**Published:** 1882-02-20

**Authors:** H. V. Ferrell

**Affiliations:** St. Louis, Mo.


### PRACTICAL OBSERVATIONS IN TYPHOID FEVER.

BY H. V. FERRELL, M. D.,

A paper read before the Tri-States Medical Society, St. Louis, at its meeting October 25th, 26th and 27th, 1881.

During the last fourteen years, it has been my fortune to be connected in one way or another with about four hundred cases of typhoid fever—for a period of near two years never being without a case under treatment. As this short paper is not the result of as thorough and complete a study of the cases as I propose, at some future time, to give them, I shall confine myself to the more salient points in the few observations I have to make.

It is taught by late writers that typhoid fever is caused by a specific typhoid fever germ—a germ that may retain its viability for an indefinite length of time—that, however bad the hygienic surroundings may be, typhoid fever will not originate *de novo*.

If the first proposition were true, we would expect to see the disease reappearing in houses that had formerly been visited by it, and might expect it to be carried by fomites. Indeed, taking up its abode in houses, and rendering them dangerous for future habitation. Or, again, assuming the correctness of the second proposition, we would not expect to find the disease in localities where it had never been known before, and affecting persons living under the best possible hygienic circumstances—persons who had never so much as seen or heard of a case of typhoid fever. In my experience, I have seen nothing to support this theory of the causation of typhoid. On the contrary, I have most generally seen it under precisely the opposite conditions. I have known typhoid fever to originate in families and in localities where it had never been known to appear before, and to confine itself, like a tornado, to a narrow, well-defined belt of country, and, after starting out upon its march, and after its direction and rate of progress were known, I have seen the time and place of its appearance correctly predicted. I have seen typhoid fever appear in the best regulated families—families living under the very best hygienic circumstances, attack one member and spare the balance; and, again, sparing none. I have seen it originate, as far as we could tell, without cause, and confine itself to those in the family, and others who visited the family on errands of mercy or friendship—none being attacked. Again, I have known intimate and free communication between patients and those living without the typhoid fever range, without any evidence whatever of contagion.

In the diagnosis of typhoid, the chief difficulty is to distinguish it from some form of malarial fever. This cannot always be done early in the disease. Typhoid is sometimes initiated by two or more well defined chills occurring periodically. Generally, however, upon close inquiry we find that the fever did not entirely subside between the chills.

When the disease is fully developed, the diagnosis is not difficult; even the laity readily recognize it. In typhoid we generally have the abdominal symptoms developed early. The rash, in my experience, is oftener absent than present. Persistent severe pain in the frontal and lumbar regions is a symptom upon which much reliance is placed, and in practice the administration of quinia for two or three days without any beneficial effect very often decides the diagnosis in favor of typhoid. A pointed tongue, red at tip and edges, abdominal tenderness, with tympanitis are usually the earliest symptoms. Epistaxis as a symptom may be discarded entirely. Sweating without a corresponding decrease of temperature may be classed among the earliest symptoms.

Some cases begin abruptly; the patient having been in usually good health up to the very moment of being stricken with a chill. Others begin so insidiously that neither the patient nor his friends have any idea that he is the victim of a serious malady until far advanced in the disease.

I call nothing typhoid fever without satisfactory evidence of intestinal lesions, at the same time admitting that similar intestinal lesions may perhaps be associated with other pathological conditions. A neglected or badly treated case of malarial fever may assume any or all of the symptoms of typhoid, except those pertaining to the intestinal lesions. Such cases it is customary with us to call typho-malarial fever—meaning by that simply a malarial fever with the typhoid symptoms superadded, and not one with intestinal lesions at all, much less lesions differing in imagination only from those of typhoid.

My acquaintance with typhus fever is too limited to justify me in speaking of it. Suffice it to say, however, that I have more than once seen cases of typhoid fever, which, occurring under the conditions under which typhus fever is said to occur, would have been pronounced typhus without any hesitation whatever. Of the complications of typhoid fever, I have seen ten cases of intestinal hemorrhage. In six of these the hemorrhage was preceded early in the disease by copious, mushy discharges from the bowels. Twice, on the appearance of such discharges, was the occurrence of hemorrhage correctly predicted, and in no case do my notes show the occurrence of such discharges without being followed by hemorrhage.

But one case of hemorrhage terminated fatally. A mild form of bronchitis was a frequent complication—pneumonia rare and never fatal. Of perforation of the intestine, two cases—both fatal, of course. In but one fatal case were there ever any serious cerebral symptoms, and that late in the disease; although in many others the delirium was alarming. The liver is said by authority never to escape more or less structural change. I have known the symptoms referable to the liver so prominent as to lead good practitioners to suppose it to be the chief seat of disease. In no case, however, have I known a diseased liver left as a legacy of typhoid fever, as determined by the subsequent history of the case. I have never known hiccough, except in cases of inflammation of the peritoneum and threatening or actual perforation.

The prognosis of typhoid fever, though as a general rule good, should always be guarded. We see cases of the mildest type continuing for a great length of time, and ending in hemorrhage, perforation, death. Others, of an apparently grave type, pursuing a short course, and ending in recovery. So, then, we may say there are no symptoms by which we can tell the extent of the intestinal lesions, or foretell what the duration or termination of the disease is going to be. In one of my fatal cases the disease had seemingly pursued a very mild course, and the patient was thought to be convalescent. He became startled in his sleep, suddenly jumped, said he felt something tear inside of him, and died of peritonitis within thirty-six hours. I have had two cases to continue as long as thirteen weeks, one apparently mild, the other grave. Both recovered. It is rare, indeed, that a case will terminate under two weeks. I have known two cases terminate fatally within a week after taking to bed, but weeks after they had been laboring under the fever.

One distinguished writer says of the temperature in typhoid, that if, from the fourth to the eleventh day of the disease, the temperature falls below 103 degrees, it is not typhoid fever. I have repeatedly seen the temperature during that period below 103 degrees—three cases, one of them fatal, in which the temperature never reached that point. On the other hand, I have seen one case in which, an hour before death, the thermometer in the axilla marked 109.8 degrees. I have seen but one case recover in which the temperature ever went above 106 degrees. In the three cases in which the temperature never rose to 103 degrees, the pulse was slow and feeble, often falling as low as forty-eight beats per minute. In one fatal case, a young clerk, who, not knowing that he was suffering from a serious malady, kept at his business for two or three weeks, trying to wear the fever off, the axillary temperature always exceeded that in the mouth by from two to three degrees.

I have seen the pulse range from 120 to 144 per minute, and the patient recover.

The London Medical Record, July 15th, 1881, calls attention to cases of typhoid fever occurring in exhausted individuals, and running their course with low temperature or without fever, but with a tendency to gangrene of the extremities. I have seen one such case, in which I amputated the thigh at its middle third—the patient made a good recovery. I have seen but two cases of typhoid during pregnancy. One recovered without aborting, and went to full time. The other is not yet determined.

The treatment of typhoid fever is quite satisfactory. While we do not possess any specific for the disease, yet the low rate of mortality is, I think, largely due to its management. The mortality in my experience barely exceeded two per cent. In the treatment there are three fundamental rules to be kept in view:

1st. Put the patient to bed early, and enjoin the most absolute rest throughout the whole course of the disease. In all cases of doubt in the diagnosis, I advise the patient to take his bed. If it is not typhoid, rest is not apt to hurt him, and if it is, it may be the very means of saving his life. In every one of my fatal cases this rule was not observed. In the two cases of death from perforation, one had been about with the fever on him for two weeks, the other three. In the one from hemorrhage, the young man tried for near three weeks to wear his fever out. I have lost no case where the patient took to bed early.

2d. Early and judicious alimentation; by early I mean within the first forty-eight hours. The aliment should be highly nutritious, easily assimilated, in a liquid form and given at regular intervals.

3d. Use drugs only to meet indications, and with a well-defined purpose, and no longer than that purpose is subserved. The German specific treatment I believe to be utterly worthless, if not worse. If the temperature runs high, use quinia and digitalis in large doses, sponge the surface freely and frequently with equal parts of whisky and water, to which may be added a little muriatic acid. To control the bowels and to correct the offensive odor of the discharges, bismuth and carbolic acid, or bismuth and liq. sod. chlorinati. For the vomiting which is sometimes very troublesome, oxalate of cerium, in 10 gr. doses, or calomel in doses of the 1-10 or 1-12 of a grain. For restlessness or sleeplessness, codeia has answered my purpose best. For intestinal hemorrhage, hypodermic injections of ergotine, or what answers just as well, Squibbs' fluid extract of ergot. For great muscular or nervous weakness I have seen tinc. nucis vomicæ produce excellent results.

Finally, I have no sort of doubt as to the utility of alcoholic stimulants early and judiciously administered.—[St. Louis Clinical Record.

